# Transparent energy-saving windows based on broadband directional thermal emission

**DOI:** 10.1515/nanoph-2023-0580

**Published:** 2024-01-09

**Authors:** Minyeol Bae, Do Hyeon Kim, Sun-Kyung Kim, Young Min Song

**Affiliations:** School of Electrical Engineering and Computer Science, Gwangju Institute of Science and Technology (GIST), Cheomdangwagi-ro 123, Buk-gu, Gwangju 61005, Republic of Korea; Department of Applied Physics, Kyung Hee University, Gyeonggi-do 17104, Yongin, Republic of Korea; Artificial Intelligence (AI) Graduate School, Gwangju Institute of Science and Technology (GIST), Cheomdangwagi-ro 123, Buk-gu, Gwangju 61005, Republic of Korea; Department of Semiconductor Engineering, Gwangju Institute of Science and Technology (GIST), Cheomdangwagi-ro 123, Buk-gu, Gwangju 61005, Republic of Korea

**Keywords:** directional emission, radiative cooling, Berreman mode, thermal management, epsilon-near-zero

## Abstract

Passive radiative cooling has emerged as a sustainable energy-saving solution, characterized by its energy-free operation and absence of carbon emissions. Conventional radiative coolers are designed with a skyward orientation, allowing for efficient heat dissipation to the cold heat sink. However, this design feature presents challenges when installed on vertical surfaces, as nearby objects obstruct heat release by blocking the cooler’s skyward view. Here, we introduce a directional radiative cooling glass (DRCG) designed to facilitate efficient heat dissipation through angular selective emission. The DRCG is constructed as a multilayer structure incorporating epsilon-near-zero materials, specifically Si_3_N_4_ and Al_2_O_3_, layered on an indium-tin-oxide thermal reflector. This innovative design restricts thermal emission to specific angular ranges, known as the Berreman mode. Additionally, the transparent layers enable a visible transmittance exceeding 84 %. Theoretical simulations validate the enhanced cooling performance of the DRCG, exhibiting a temperature reduction of over 1.5 °C compared with conventional glass in hot urban environments characterized by a nearby object temperature exceeding 60 °C and a sky view factor of 0.25. Furthermore, outdoor experiments demonstrate that employing the DRCG as a window enhances space-cooling performance by ∼1.5 °C. These findings underscore the potential of transparent energy-saving windows in mitigating the urban heat island effect.

## 1 Introduction

Addressing global climate change and environmental issues, including air pollution and ozone depletion, requires a worldwide reduction in energy consumption and CO_2_ emissions. In this context, energy-efficient cooling technology has gained prominence, as traditional cooling systems account for ∼20 % of global energy consumption and contribute to the depletion of fossil fuels [1]. Passive radiative cooling has emerged as a promising strategy due to its energy-free and zero-carbon emission characteristics[2–11]. Such a strategy requires high emissivity within the long-wave infrared (LWIR) atmospheric window, allowing passive heat dissipation into the cold outer space. This innovative approach has opened up research opportunities in various domains, including solar cells [12], [13], fluids [14], electronic devices [15], [16], vehicles [17], [18], and buildings [19], [20]. Most passive radiative coolers are designed with a skyward orientation to enhance the heat dissipation to the cold heat sink. However, this design characteristic presents challenges when applied to vertical surfaces, such as windows and building exteriors. The presence of nearby objects, including trees, pedestrians, and other buildings, obstructs the skyward view of wall-mounted coolers, impeding heat release into the cold outer space. Additionally, these neighboring objects emit heat that is undesirably absorbed by the coolers. Consequently, installing conventional coolers on walls becomes a challenge for efficient heat dissipation, particularly in densely populated areas (*e.g.*, urban areas).

Alternatively, directional thermal emitters, which restrict thermal emission to specific angular ranges, offer a solution to circumvent heat exchange with nearby obstacles when mounted on walls. Numerous studies on directional thermal emitters have introduced various structures, including surface plasmon polariton-based gratings [21–24], metamaterials [25], and metasurfaces that support the Brewster angle [26]. However, these approaches often entail complex fabrication processes [27]. In contrast, recent research has employed epsilon-near-zero (ENZ) materials on a reflector to achieve directional thermal emissions based on a Berreman mode that exhibits strong absorption at a specific incident angle for p-polarized light [28]–[30]. The ENZ materials (*e.g.*, SiO_2_, Si_3_N_4_, Al_2_O_3_, and TiO_2_) intrinsically possess narrowband characteristics within the LWIR region (*i.e.*, 8–13 μm), resulting in narrowband directional emission. To achieve broadband directional emission, researchers have introduced multilayered ENZ materials that generate broadband Berreman modes [28], [29]. These configurations exhibit gradient ENZ behavior at different wavelengths, enabling broadband directional emission [28]. Despite progress in developing broadband directional thermal emission, previous studies lacked a comprehensive theoretical model for analyzing the cooling performance of these emitters when installed on vertical surfaces. Furthermore, the employment of metal mirrors (*e.g.*, Au and Al) on ENZ layers [28], [29] results in opaque to visible light, making them unsuitable for window applications and limiting their practical utility.

Here, we introduce a directional radiative cooling glass (DRCG) that exhibits broadband directional thermal emission and visible transparency. The DRCG consists of double-layered ENZ materials comprising Si_3_N_4_ and Al_2_O_3_ on an ITO-coated glass. Notably, the ITO-coated glass serves as a thermal reflector owing to its high reflectance (∼93 %) in the LWIR region. Si_3_N_4_ and Al_2_O_3_ possess distinct ENZ wavelengths, specifically 8.8 and 10.7 μm, respectively, within the LWIR range. Thus, the combination of ENZ layers and ITO supports broadband Berreman modes, facilitating broadband directional thermal emission. Moreover, the transparent layers (*i.e.*, Si_3_N_4_, Al_2_O_3_, and ITO) ensure a high level of visible transparency (∼84 %), rendering it suitable for use in windows. To validate the DRCG’s cooling performance, we have developed a comprehensive theoretical model that considers the presence of nearby objects surrounding the wall-mounted cooler. Our theoretical simulations clearly demonstrate the superior cooling performance of the DRCG, with a temperature reduction exceeding 1.5 °C compared with conventional glass (C-glass) in hot urban environments. These environments are characterized by elevated temperatures of nearby objects exceeding 60 °C and typical urban building configurations featuring a sky view factor of approximately 0.25 [31]. Furthermore, outdoor experiments demonstrate that employing the DRCG as a window enhances the space cooling performance by approximately 1.5 °C when compared with C-glass. This underscores the potential of our proposed DRCG as an energy-saving window capable of mitigating the urban heat island effect by minimizing heat exchange with adjacent obstacles.

## 2 Results and discussions

### 2.1 DRCG for energy-saving window

**Figure 1:**
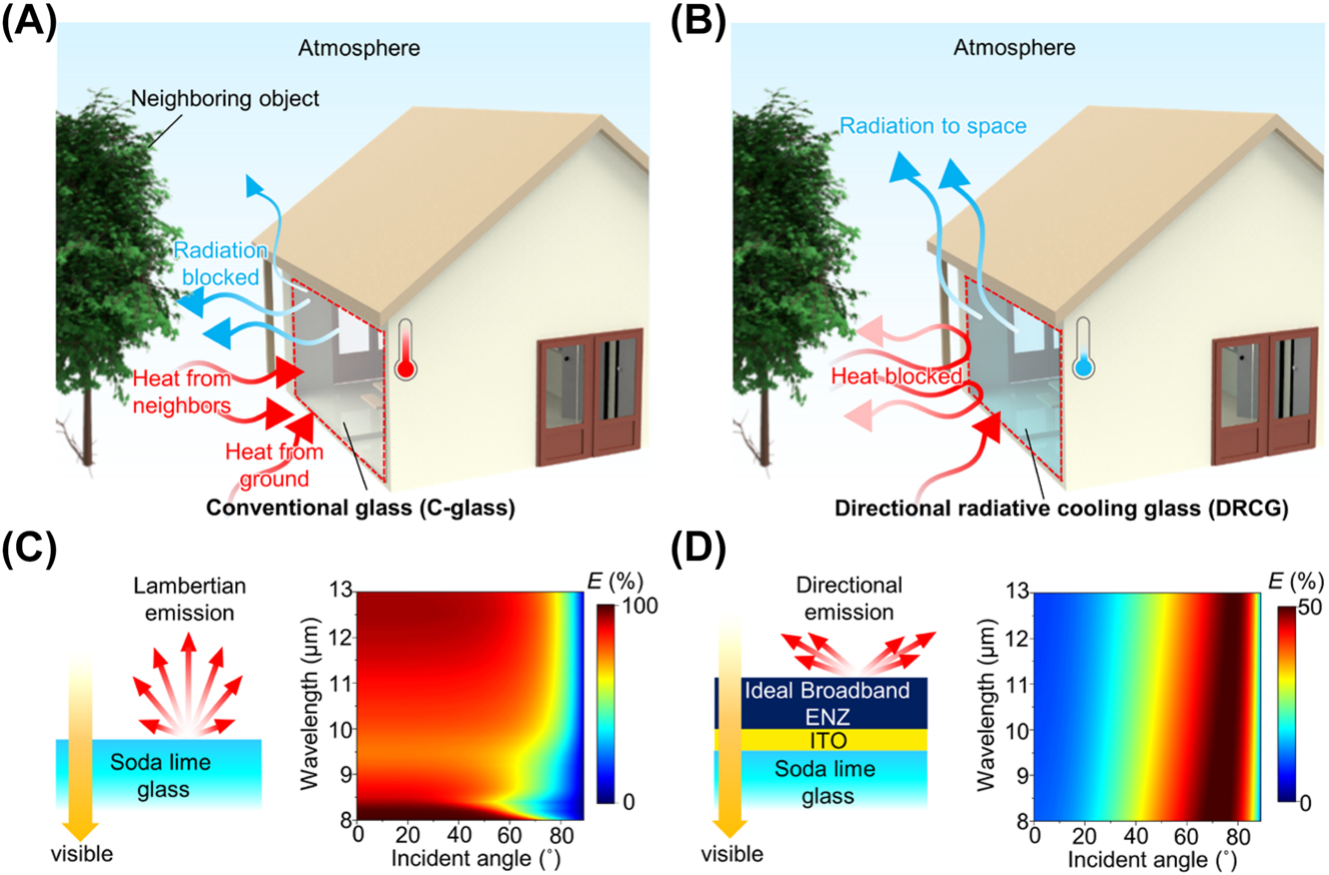
DRCG for energy-saving window. (A, B) Schematic of the heat-exchanging process between the surrounding objects (*i.e.*, neighboring objects and ground) and glass windows for (A) conventional glass (C-glass) and (B) the directional radiative cooling glass (DRCG). (C, D) Visible and thermal optical properties of the (C) C-glass and (D) ideal DRCG.

Figure 1A illustrates the heat exchange process between the C-glass window and its surrounding obstacles, including neighboring objects and the ground. The C-glass exhibits strong Lambertian emission in the thermal spectrum, facilitating substantial thermal radiation into its surroundings. However, the presence of nearby obstacles blocks the heat dissipation from the C-glass, and these neighboring objects also emit a significant amount of heat toward the C-glass. This mutual heat exchange process leads to heat being trapped between the C-glass and neighboring obstacles, increasing temperature. In contrast, the DRCG minimizes heat absorption from neighboring objects due to its lateral thermal emission, as depicted in Figure 1B. This directionality reduces heat gain from adjacent obstacles while ensuring efficient heat dissipation toward the skyward direction. Consequently, the DRCG window maintains a lower temperature than the C-glass window by preventing heat gain from nearby objects. This effect is particularly pronounced in densely populated scenarios, such as urban areas, where neighboring objects block a significant portion of the radiation from the window. Therefore, the DRCG holds the potential to serve as an energy-saving window capable of mitigating the urban heat island effect by minimizing heat exchange with neighboring objects.

When neighboring objects face the glass window on the wall, the glass predominantly absorbs heat from these adjacent obstacles at low incident angles (<60°) where the neighboring objects are mostly located. Thus, a high thermal emissivity of the glass at this ranges (<60°) causes substantial heat absorption from nearby objects, resulting in heating (Figure S1). The C-glass exhibits strong Lambertian emission in the LWIR range and high transmittance (∼90 %) in the visible region, as demonstrated in Figures 1C and S2. The high emissivity of the C-glass at low incident angles (<60°) contributes to the entrapment of heat between neighboring objects and the glass itself. In contrast, the DRCG avoids heat exchange with neighboring objects owing to its directional emissivity that is limited to high incident angles (>60°), lowering window temperature. An ideal DRCG exhibits pronounced directionality in the thermal region while maintaining prominent transmittance in the visible range, as observed in Figure 1D. The ideal DRCG’s structure comprises ideal broadband ENZ, ITO, and soda lime glass layers. Including an ideal broadband ENZ material that covers the entire LWIR region while preserving visible transparency sets an upper limit for the DRCG’s cooling performance. The ITO layer serves as a visibly transparent and mid-infrared (MIR) mirror, with significant transmittance (∼84 %) in the visible region and MIR reflectance (∼93 %), as depicted in Figure S3. The ideal broadband ENZ layer on the MIR mirror (*i.e.*, ITO) generates prominent directional emission across the entire LWIR region for p-polarized light based on broadband Berreman modes. Thus, the DRCG achieves high visible transparency (>84 %, see Figure 3B) and broadband directional emission in the LWIR region, suitable for energy-saving window applications. The refractive indices of the soda lime glass and ideal broadband ENZ material appear in Figure S4.

### 2.2 Design rules of the DRCG

**Figure 2:**
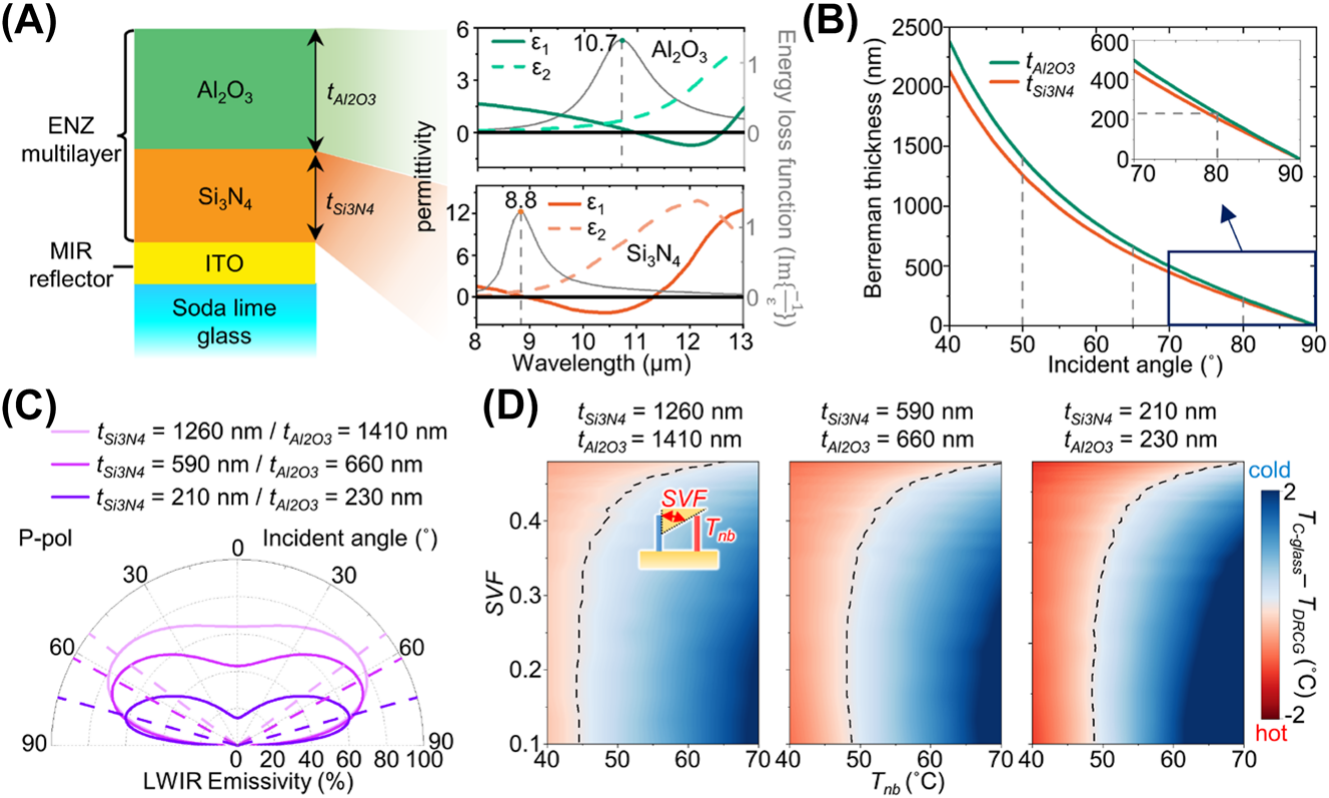
Design rules of the DRCG. (A) Structure of the DRCG (left) and permittivity of the materials (right). Dashed lines indicate the ENZ wavelength of the materials. (B) Calculated Berreman thickness of Al_2_O_3_ and Si_3_N_4_. (C) Calculated average emissivity over the LWIR range of three structures with different thicknesses as a function of the incident angles in p-polarization. The dashed lines indicate the peak angles of each structure. (D) Simulated temperature difference between the three structures and the C-glass with different neighboring object temperatures (*T*_nb_) and sky view factors (SVFs).

Figure 2A presents a structure of our DRCG with the ENZ wavelengths of ENZ materials. The DRCG comprises a double layer of ENZ materials (*i.e.*, Si_3_N_4_ and Al_2_O_3_) and a MIR reflector (*i.e.*, ITO) on a soda lime glass substrate. The optical constants of Al_2_O_3_, Si_3_N_4_, and ITO, obtained from previously published research [32–34], are shown in Figure S5. Si_3_N_4_ and Al_2_O_3_ possess ENZ wavelengths of 8.8 and 10.7 μm, respectively. Both ENZ wavelengths fall within the LWIR range, exhibiting a distinct difference between them. The combination of these materials collectively covers a significant portion of the LWIR region. Consequently, the double layer of ENZ materials on top of the ITO layer enables the DRCG to achieve broadband directional thermal emission, owing to the Berreman mode of each layer [28].

When considering a single layer of ENZ material on a mirror, the thickness of ENZ layer that maximizes emissivity at a specific incident angle, known as the Berreman thickness, can be determined using the generalized Fresnel equation [35]:

(1) $$t_{B}=\frac{\lambda }{2\pi }\frac{\cos\theta }{\sin^{2}\theta }{\left(\text{Im}\left\{\frac{-1}{\varepsilon }\right\}\right)}_{\max}^{-1},$$

where *λ* is the ENZ wavelength, *ε* is the permittivity of the ENZ material, and *θ* is the incident angle. The calculated Berreman thickness of Si_3_N_4_ and Al_2_O_3_ as a function of incident angles are presented in Figure 2B. As appears in Equation (1), the calculated Berreman thickness decreases with elevating the incident angle. Consequently, the control of ENZ film thickness enables the regulation of emissivity peak angles. Figure 2C illustrates the average emissivity across the LWIR range in p-polarization for three different structures that incorporate double-layered ENZ materials with varying thicknesses. These structures exhibit directional thermal emission with distinct emissivity peak angles at 53°, 62°, and 75°, respectively, arranged in order of decreasing total thickness. Notably, all three structures feature emissivity peaks near 8.8 and 10.7 μm, corresponding to the ENZ wavelengths of Si_3_N_4_ and Al_2_O_3_, respectively (as depicted in Figure S6). This result confirms that our designed DRCGs represent broadband directional emission, covering a substantial portion of the LWIR spectrum owing to the Berreman mode of each layer. Increasing the total layer thickness above these structures induces transverse optical phonon polariton (TO) resonance, resulting in non-directional thermal emission [29].

Figure 2D presents the calculated cooling temperature differences between three distinct structures and the C-glass, with respect to varying neighboring object temperatures and sky view factors (SVFs). The SVF quantifies the fraction of the visible sky for the object [36]. To determine the SVF, we utilized the equation established in prior research, as follows [37]:

(2) $$\text{SVF}=\frac{1}{\pi R^{2}}\int_{S_{V}}\cos\left(\theta \right)\text{d}S,$$

where *R* is the radius of a hemisphere, *S*_*V*_ is the hemispheric section of the visible sky, and *θ* is the zenith angle. The SVF was determined at the central point of the glass, with a ground temperature assumed to be 30 °C. Further details regarding the methodology used for calculating cooling performance can be found in Section 2.4.

In Figure 2D, the blue regions indicate conditions where the DRCG exhibits lower temperatures than the C-glass, while the red areas represent the opposite scenario, where the DRCG exhibits higher temperatures. The dashed lines mark situations where the DRCGs and C-glass are the same temperature, denoted as *T*_DRCG_ = *T*_C-glass_. In scenarios where neighboring obstacles surround the wall-mounted glass, two primary factors influence cooling performance: (1) the radiation power of the glass and (2) heat absorption from neighboring objects. A higher building density tends to reduce the SVF because of neighboring obstacles, such as other buildings. Thus, low SVF indicates the obstructed window’s view of the sky, impeding the heat dissipation from the glass. Conversely, elevated SVFs (>0.47) result in an expanded sky view for the cooler, reducing the heat supply from obstacles to the coolers. When neighboring object temperatures are below 50 °C, the heat emission from these objects to the glass is relatively minor, as this temperature range is closer to the ambient temperature. Consequently, in such scenarios, the radiation power of the emitter becomes more significant than absorption from neighboring objects. The radiation power is directly proportional to the total thickness of the structures, with thicker structures increasing the total emissivity. Thus, the thickest DRCG exhibits the best cooling performance at low neighboring object temperatures (<50 °C) and high SVFs (>0.47). In contrast, at elevated neighboring object temperatures (>50 °C) and low SVFs (<0.47), a substantial thermal emission from neighboring objects reaches the glass through a broad angle. In such cases, a low emissivity at low incident angles is essential to prevent undesired heat absorption from the surroundings. Consequently, the thinnest DRCG design demonstrates enhanced cooling performance at elevated neighboring object temperatures of >60 °C and SVFs of <0.47. Considering the high building density observed in urban areas (SVF = ∼0.25), the thinnest DRCG structure proves to be optimal for urban regions during hot summer days (*T*_nb_ > 60 °C).

### 2.3 Optical measurements of the fabricated DRCG

**Figure 3:**
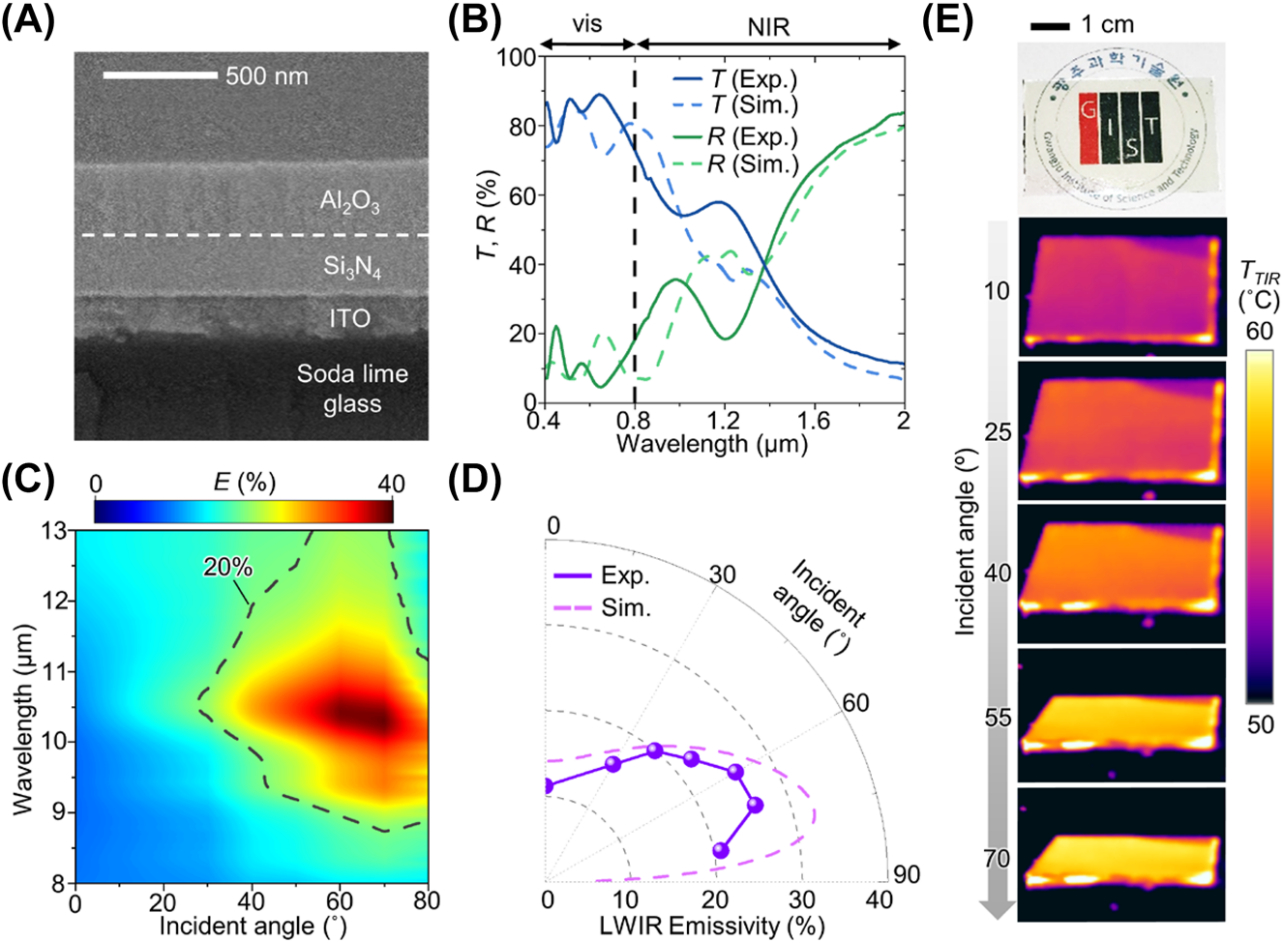
Optical measurements of the fabricated DRCG. (A) SEM image of a fabricated DRCG. (B) Measured and simulated transmittance/reflectance over the vis–NIR region of the fabricated DRCG. (C) Variation in the measured emissivity spectrum of the fabricated DRCG with incident angles under unpolarized conditions. (D) Variation in the measured and simulated average emissivity across the LWIR range of the fabricated DRCG with the incident angles under unpolarized conditions. (E) Photograph of the fabricated DRCG (top) and thermal images at different incident angles of the fabricated DRCG.

As appears in Figure 3A, a scanning electron microscopy (SEM) image shows the fabricated DRCG. The ENZ materials of Al_2_O_3_ and Si_3_N_4_ are deposited on a commercial ITO-coated glass (*i.e.*, ITO coated on soda lime glass) with the desired thickness. Detailed information regarding the fabrication process can be found in the Methods section. The fabricated DRCG exhibits high transmittance (∼84 %) in the visible region, as depicted in Figure 3B. This is attributed to the use of transparent materials, including Al_2_O_3_, Si_3_N_4_, and ITO-coated glass. However, the commercial ITO-coated glass, optimized for electronic devices, exhibits a slight solar absorption (∼6 %), leading to a lower visible transmittance compared with the C-glass (∼90 %). The transmittance enhancement can be achieved through control of the deposition conditions and thickness, potentially improving the visible transmittance of the ITO to ∼92 % [38]. Therefore, reducing ITO thickness can enhance the visible transmittance compared with commercial ITO glass, making it suitable for window applications. Furthermore, by incorporating a near-infrared (NIR)-reflective structure, such as the insulator-metal-insulator layer, we can effectively reduce the NIR transmittance of the DRCG from 30 % to 20 % while minimizing the visible transmittance loss (76 %), as shown in Figure S7. Incorporating a NIR-reflective structure further improves the cooling performance in real-world scenarios by blocking the solar radiation.

To validate the directional characteristics of our fabricated DRCG, the emissivity spectrum was measured under unpolarized light, as shown in Figure 3C. The fabricated DRCG exhibits emissivity peaks around 8.8 and 10.7 μm, corresponding to the ENZ wavelengths of Si_3_N_4_ and Al_2_O_3_, respectively. The dashed line in Figure 3C represents an emissivity of 20 % in unpolarized light, highlighting that the elevated emissivity (>20 %) range is predominantly located at high incident angles within the LWIR range. This observation indicates the broadband directional thermal emission features of the fabricated DRCG. The measured emissivity at different incident angles closely aligns with the simulated results, as depicted in Figure 3D. Within the angular range from 60° to 70°, the average emissivity of the fabricated DRCG exceeds 25 %, while it remains at only ∼11 % at 0°. Since the multilayer structure featuring two ENZ materials cannot cover the entire LWIR region, the average emissivity at high incident angles (>60°) of the fabricated DRCG is less than that of the ideal scenario (*i.e.*, 50 %). Despite this lower emissivity, the simulation results demonstrate that the average emissivity of 25 % enables lowering the temperature than ambient temperature (Figure S8). The incorporation of a dielectric gap, such as ZnSe, that generates a Fabry–Pérot resonance can enhance the average emissivity closer to the near-ideal scenario [29], while maintaining visible transmittance of ∼70 %, as demonstrated in Figure S9. With the addition of the dielectric gap, the calculated cooling temperature increases by 20 % compared with the original DRCG at a neighboring object temperature of 60 °C and an SVF of 0.45.

Figure 3E presents optical and thermal images of the fabricated DRCG. Although the fabricated DRCG exhibits a bright yellow color due to the slight absorption of the visible spectrum by the ITO, underlying patterns are discernible due to the high transmittance of the fabricated DRCG. The fabricated DRCG was heated to 60 °C by a hot plate, and its thermal radiation was captured at various incident angles (*i.e.*, 10, 25, 40, 55, and 70°) using a thermal camera. The directional thermal emission of the fabricated DRCG enlarges the amount of thermal radiation with the increment of incident angles. Consequently, the apparent temperatures of the fabricated DRCG, as seen in the thermal images, rise according to the increasing incident angles. These thermal images effectively demonstrate the directional thermal emission property of our fabricated DRCG.

### 2.4 Theoretical surface cooling performance of the DRCG and C-glass

**Figure 4:**
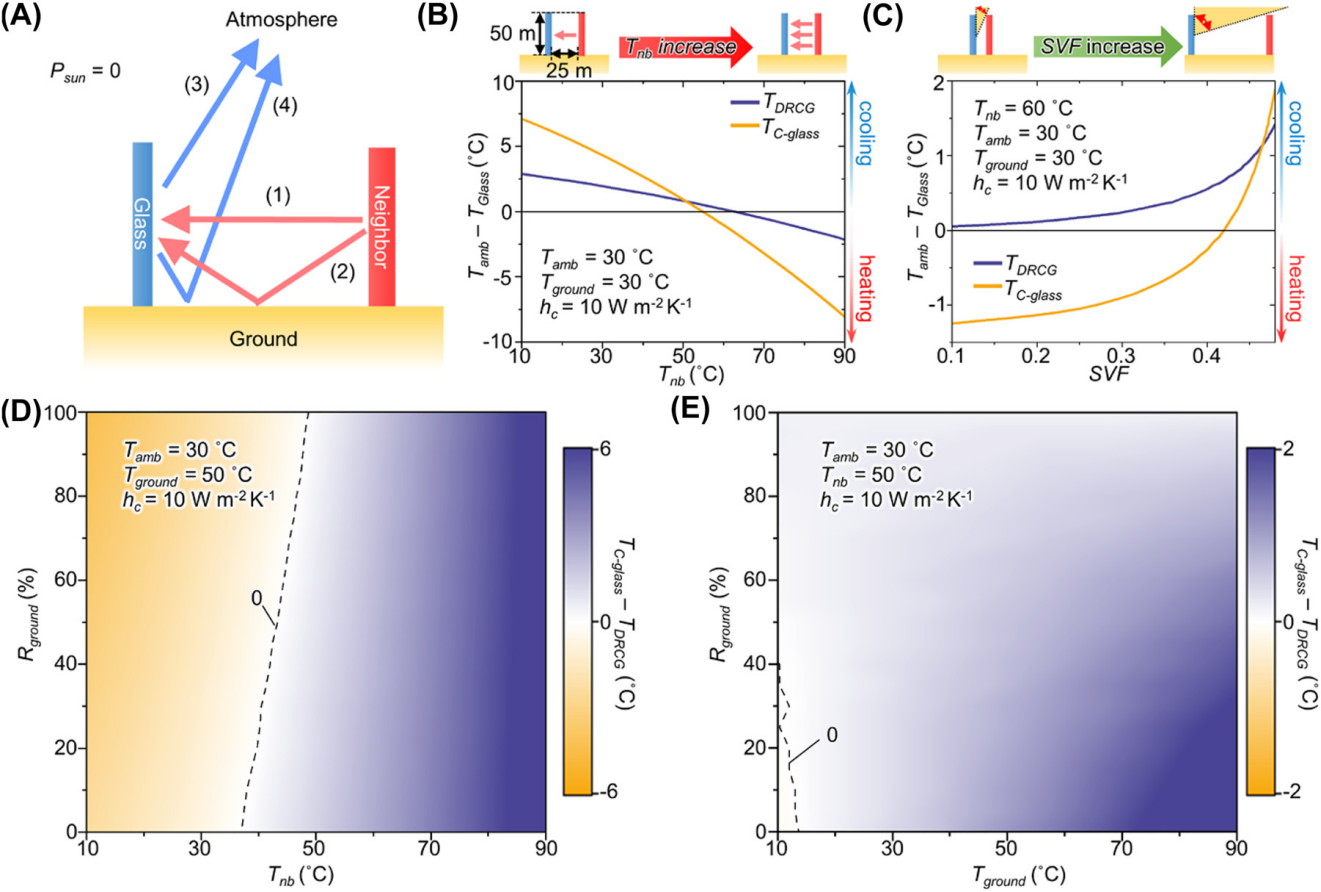
Theoretical surface cooling performance of the DRCG and C-glass. (A) Schematic of the heat exchange process between neighboring objects and the glass with four different thermal radiation paths. (B, C) Calculated cooling temperature of the DRCG and C-glass as a function of the (B) neighboring object temperature and (C) sky-view factor (SVF). (D) Simulated surface temperature difference between the DRCG and C-glass as a function of the neighboring object temperatures (*T*_nb_) and thermal ground albedo (*R*_ground_). (E) Simulated temperature difference between the DRCG and C-glass with different ground temperatures (*T*_ground_) and thermal ground albedo.

**Figure 5:**
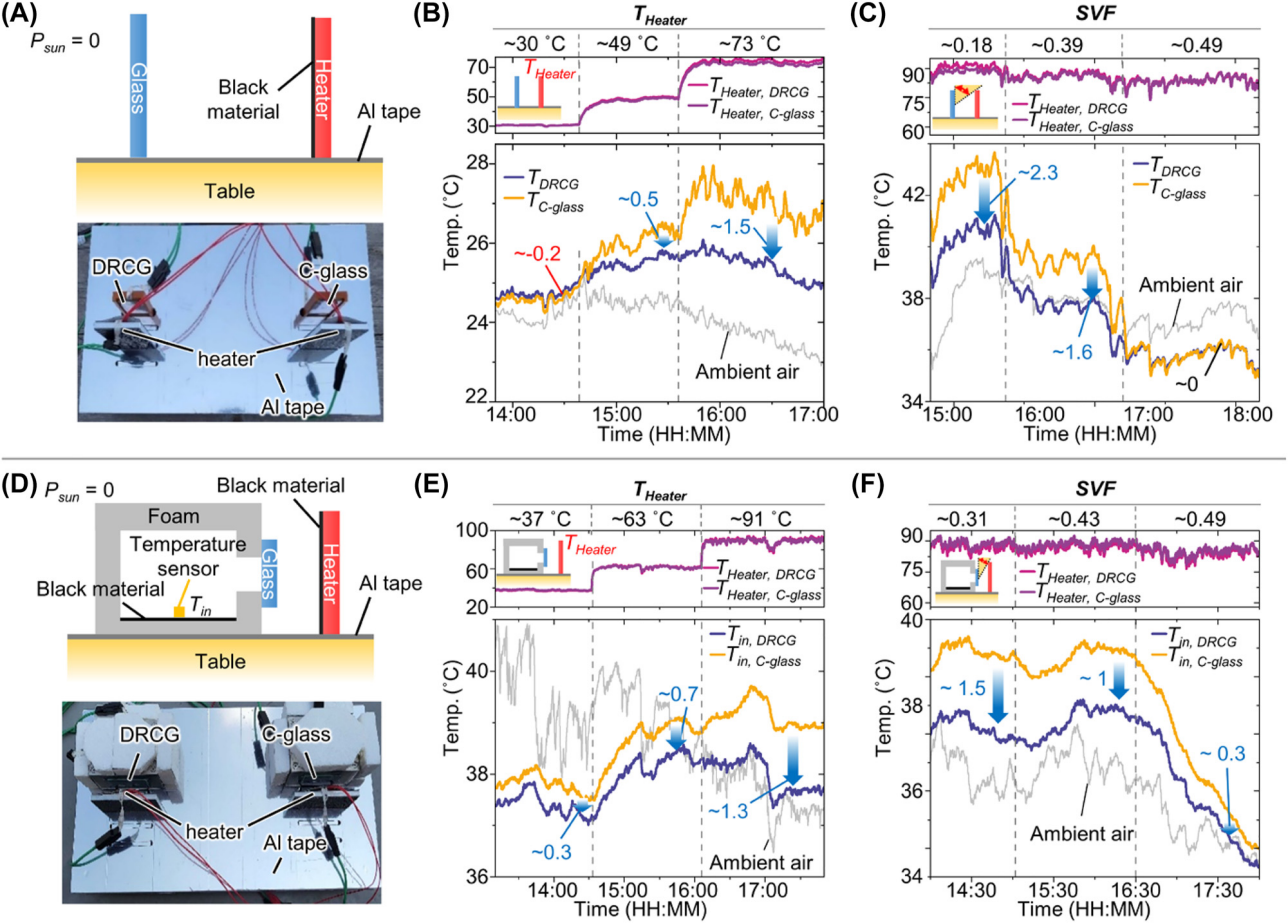
Surface and enclosure cooling measurements of the DRCG and C-glass. (A) Schematic and photograph of the surface cooling measurement setup. (B, C) Experimental result of surface temperatures of the DRCG and C-glass under conditions of varying (B) neighboring object temperatures and (C) SVFs. (D) Illustration and optical image of the enclosure cooling measurement setup. (E, F) Experiment result of the enclosure temperature of DRCG and the C-glass under the conditions of (E) different neighboring object temperature and (F) different SVFs.

Figure 4A illustrates the heat exchange process between a neighboring object and the glass. Heat transfer from the neighboring object to the glass predominantly transpires through two distinct paths: direct thermal emission (path 1) and reflection from the ground surface (path 2). These processes result in the heat absorption by the glass. Conversely, the glass dissipates heat through two different paths: direct emission into the atmosphere (path 3) and emission after reflection off the ground (path 4). These emissions contribute to heat dissipation. For the sake of simplicity, we did not account for other optical pathways. We employed a modified thermal equilibrium equation to assess the cooling performance of both the DRCG and the C-glass while considering the presence of neighboring objects:

(3) $$\begin{array}{ll} & P_{\text{rad}}\left(T_{\text{sample}}\right)-P_{\text{Sun}}-P_{\text{amb}}-P_{\text{nb}}-P_{\text{ground}}\\ & \;+P_{\text{non }-\text{ rad}}=0, \end{array}$$

where *P*_rad_(*T*_sample_) is the radiative power per unit area of the sample, *P*_Sun_ is the solar power per unit area, *P*_amb_ is the absorbed power per unit area from ambient air, *P*_nb_ is the absorbed power per unit area from the neighboring objects, *P*_ground_ is the absorbed power from the ground, and *P*_non-rad_ is the non-radiative heat exchange power per unit area (*e.g.*, conduction and convection). *P*_amb_, *P*_nb_, and *P*_ground_ are obtained by considering the angular emissivity of the sample, ambient air, neighboring objects, and ground. In our calculations, we intentionally excluded the solar power term (*i.e.*, *P*_Sun_ = 0) to primarily assess the influence of directional thermal emission on cooling performance (Figure 4). Consequently, all outdoor experiments, depicted in Figure 5, were conducted under shaded conditions to eliminate solar illuminations. Our specific focus was on the side of the glass that directly faces the neighboring object in the simulation. Additional details regarding the calculation conditions can be found in the Methods section and observed in Figures S10 and 11.

Figure 4B illustrates the computed cooling performance of both the DRCG and C-glass as we varied the temperatures of neighboring objects. The glass and neighboring object heights were fixed at 50 m, corresponding to the height of a 20th-floor apartment [39]. The distance between the glass and the neighboring object was set at 25 m to simulate urban conditions, aligning with the regulated minimum spacing for 20th-floor apartments in Korea [40]. In this configuration, the SVF is 0.25. We employed a cylindrical-shaped neighboring object to represent equal distances between the glass and neighboring objects along horizontal directions. A thermal ground albedo of 90 % was applied to primarily investigate the influence of neighboring objects on cooling performance by excluding ground emission.

As demonstrated in Figure 4B, when the neighboring object temperature is lower than 50 °C, the C-glass outperforms the DRCG. This outcome arises because, given the proximity of the neighboring object temperature to the ambient temperature, the heat absorption from neighboring objects (via paths 1 and 2) is relatively minor compared with the heat dissipation from the glass to the outer space (via paths 3 and 4). In such circumstances, the C-glass exhibits superior cooling performance due to its higher total emissivity compared with the DRCG, enabling greater heat dissipation. Conversely, when beginning with a neighboring object temperature of 60 °C, the DRCG demonstrates better cooling performance (>1.5 °C) than the C-glass. This is because the C-glass absorbs a substantial amount of heat from neighboring objects, owing to its high emissivity at low incident angles, especially in the vicinity of neighboring objects (<60°). In contrast, the DRCG effectively prevents heat gain from nearby objects, benefiting from its low emissivity at low incident angles (<60°). This result suggests that the DRCG exhibits enhanced cooling capability on hotter days compared with the C-glass, particularly since the temperature of the building wall exceeds 60 °C under direct sunlight [41], [42].

Furthermore, we conducted an analysis of the cooling performance of both the DRCG and the C-glass under varying SVF conditions, as depicted in Figure 4C. A low SVF signifies that the neighboring object substantially obstructs the cooler’s view of the sky, implying a high building density area. Hence, as the SVF decreases, heat absorption from nearby objects intensifies, while heat emission to the sky becomes impeded, exacerbating the urban heat island effect. Under conditions of high SVF (>0.47), the C-glass outperforms the DRCG in terms of cooling performance. The expanded field of view of the C-glass towards the sky facilitates more effective heat dissipation, owing to its higher total emissivity compared to the DRCG. Conversely, when the SVF decreases (<0.47), the DRCG surpasses the C-glass in cooling performance. The limited field of view of the cooler, coupled with substantial heat absorption from nearby objects, obstructs the heat dissipation of the C-glass. In contrast, the DRCG avoids heat absorption from neighboring objects and efficiently releases heat to the sky. To compare the SVF values with practical SVF, we calculated the SVF of the ground in our simulation conditions (Figure S12). After the recalibration, the window SVF of 0.47 is converted to the ground SVF of 0.90, which is higher than the SVF of cities, varying between 0.23 and 0.84 [43]. Consequently, in most urban areas, employing the DRCG as a window would contribute to energy savings for cooling compared to the C-glass.

Furthermore, we considered the influence of neighboring objects and the ground while varying the temperatures of the surroundings, including neighboring objects and the ground, as well as the thermal ground albedo (see Figure 4D and E). In these figures, the blue region signifies conditions where the DRCG exhibits superior cooling performance to the C-glass, while the yellow area represents the opposite scenario. To account for practical situations, we set the ground and neighboring object temperatures to 50 °C since they can exceed this temperature in hot weather [41], [44]. As shown in Figure 4D, DRCG demonstrates better cooling performance at higher neighboring object temperatures (>40 °C). In addition to neighboring objects, the ground also emits heat towards the glass, thereby impeding cooling. The thermal ground albedo obstructs ground emissions while increasing thermal radiation from neighboring objects along path 2. However, at low neighboring temperatures (<50 °C), the total heat flux from the ground (via ground-cooler path) exceeds that from the neighboring object (via neighboring object-ground-cooler path), leading to increased heat emission to the glass with decreasing ground albedo. Furthermore, the C-glass absorbs more substantial heat gain from the ground compared to the DRCG, primarily due to its higher total emissivity, which results in increased heat absorption. Consequently, as thermal albedo decreases at the same neighboring object temperature, the DRCG outperforms the C-glass in terms of cooling temperature. Furthermore, commencing from the temperature indicated by the dashed line in Figure 4E, as the ground temperature increases, the DRCG demonstrates superior cooling performance to the C-glass due to its lower emissivity at low incident angles. Considering practical conditions, such as high neighboring object and ground temperatures (>50 °C) on sunny days, the calculated results suggest that the DRCG would exhibit better cooling performance than the C-glass under the typical thermal ground albedo below 30 % [45]. Therefore, DRCG affords improved cooling performance than the C-glass in the city region in hot summer days. Similar to the DRCG, the low-emissivity (Low-E) glass also inhibits heat from the surroundings due to its low emissivity across whole incident angles. However, it falls short of outperforming the DRCG in terms of cooling efficiency at practical ground temperatures (*T*_ground_ < 70 °C) [46], attributed to its limited heat dissipation capacity (Figure S13). In addition, the silver layer in the low-E glass leads to a loss in visible transmittance, resulting in a lower visible transmittance of 76 % compared to our DRCG (84 %) (Figure S13). These findings highlight the enhanced applicability of the DRCG as an energy-saving window compared to the low-E glass.

### 2.5 Surface and enclosure cooling measurements of the DRCG and C-glass

Figure 5A illustrates both the schematic and photograph of the surface cooling measurement setup. To mimic building exteriors with high emissivity (>85 %) [47], we employed heaters covered in black material. This black material serves as a source of blackbody radiation from the exterior wall while the heater controls the wall temperature. Additionally, we applied Aluminum tape to the table to prevent ground emissions, allowing us to primarily investigate the influence of neighboring objects on cooling performance. We measured the surface temperature of two different types of glass (*i.e.*, DRCG and C-glass) under various heater temperatures (Figure 5B). To compare the cooling performance of these two glasses, we calculated the temperature difference between the C-glass and the DRCG (Δ*T* = *T*_C-glass_ – *T*_DRCG_). When the heater temperature is approximately 30 °C, the temperature of the C-glass is slightly lower than that of the fabricated DRCG (Δ*T* = ∼0.2 °C), primarily due to the relatively minor impact of neighboring objects. However, as the heater temperature surpasses 49 °C, the temperature of the fabricated DRCG becomes lower than that of the C-glass (Δ*T* > 0.5 °C) because the heated neighboring objects begin to affect the glasses, consistent with the calculated results shown in Figure 4B. Figure 5C presents the measurement of surface cooling temperatures corresponding to different SVFs. Figure 5C presents the measurement of surface cooling temperatures corresponding to different SVFs. In certain instances, such as rooftop solar panels and dark-colored bricks, the neighboring object temperatures can soar beyond 80 °C when exposed to direct solar radiation [48], [49]. To evaluate the DRCG’s cooling capabilities under such intense conditions, we monitored the temperature of the surface and enclosures while elevating the heater’s temperature approximately by 90 °C. At a low SVF (∼0.18), our fabricated DRCG exhibits a lower temperature than the C-glass (Δ*T* = ∼2.3 °C) because our DRCG effectively prevents thermal absorption from the heater. As the SVF increases, a reduction in heat flux from the heater is observed, resulting in a smaller temperature difference between the two glasses, as shown in the calculated results. The absolute values of cooling temperatures slightly differ from those in Figure 4. This difference can be attributed to measurement factors, such as the planar shape of the heater and the variability in the non-radiative heat exchange coefficient (*h*_*c*_). Nevertheless, the measured results confirm the superior cooling performance of the DRCG in hot and densely populated conditions.

Figure 5D presents the schematic and photograph of the enclosure temperature measurement setup. In our setup, both the DRCG and C-glass samples were installed as windows in a miniature house. We covered the house with foam to minimize conduction effects during the measurement. The bottom black material of the house simulates furniture (*e.g.*, a leather sofa and the floor). We measured the temperature of this bottom black material as the inside temperature. At a low heater temperature of ∼37 °C, representing the neighboring object, a slight temperature difference within the enclosures (Δ*T* = ∼0.3 °C) between the DRCG and the C-glass was observed (Figure 5E). However, as the heater temperature exceeded 63 °C, the DRCG exhibited improved cooling performance relative to the C-glass (Δ*T* > 0.7 °C). Since the energy usage increases by ∼10 % to lower the inner temperature by 1 °C [50], [51], these results demonstrate that employing the DRCG as a window can sufficiently contribute to energy savings for cooling compared to the C-glass. Like the trend observed in Figure 5C, the inner temperature difference between the DRCG and C-glass increased as the SVF decreased (Figure 5F). At an SVF value of 0.31, the DRCG demonstrated superior space cooling performance compared with the C-glass (∼1.5 °C). As the SVF increased beyond 0.43, this temperature difference decreased (Δ*T* < 1 °C). The use of soda lime glass as a substrate for the DRCG enables heat absorption from the inner space, while the emission from the top side facilitates the transfer of heat to the cold heat sink [17], [18]. This combination allows for efficient heat dissipation within the enclosure, in addition to the surface. These results confirm that our fabricated DRCG, when used as a window in densely built urban areas, has the potential to effectively reduce enclosure temperatures on hot summer days typically characterized by low SVF and elevated neighboring object temperatures.

For a realistic assessment of cooling performance, it is essential to include the effects of solar irradiation in both our simulations and outdoor measurements. Therefore, we incorporated solar irradiation (*i.e.*, *P*_Sun_ > 0) in both simulation and measurements. Additional details regarding the *P*_Sun_ appear in the Methods section. The simulation results demonstrate that the DRCG consistently provides superior cooling performance compared to the C-glass even under sunlight in hot summer (*T*_nb_ > 60 °C) and in densely populated urban (SVF < 0.36) conditions (Figure S14). The slight reduction in the DRCG’s cooling performance is attributed to slight solar absorption from the higher visible absorption of the DRCG (∼6 %) compared to that of C-glass (∼1 %) due to the commercial ITO-coated glass used in the DRCG, optimized for electronic devices. The solar absorption of the DRCG can be mitigated by decreasing the ITO thickness, as explained in Section 2.3. Furthermore, the DRCG outperforms the C-glass under varying solar irradiation and ambient temperature (Figure S15).

For practical validation under solar irradiation, we conducted day-to-night outdoor measurements of the surface and enclosure temperatures (Figures S16 and 17). The measured surface and enclosure temperature of the DRCG were consistently lower (>1 °C) than that of the C-glass even under varying solar irradiation, indicating superior cooling performance of the DRCG in the daytime. At daytime, the temperature difference (*T*_C-glass_–*T*_DRCG_) depended on the neighboring temperature variation caused by solar irradiation because the heat flux from the neighboring object (*P*_nb_) was larger than the solar absorption (*P*_Sun_). At night-time, *T*_C-glass_–*T*_DRCG_ increases owing to lower ambient temperature (<10 °C), consistent with simulation results (Figure S15). Additionally, the enclosure temperature measurements further confirm that the DRCG provides superior enclosure cooling performance (>1 °C) compared to the DRCG, indicating the DRCG’s effectiveness in dissipating enclosure heat even under sunlight (Figure S17D).

## 3 Conclusions

As a strategy to enhance the cooling performance of the coolers on the vertical surfaces, the DRCG shows directional thermal emission and prominent visible transmittance by employing Al_2_O_3_ and Si_3_N_4_ on ITO-coated glass. Owing to the high LWIR reflectance of ITO, our fabricated DRCG exhibits broadband directional thermal emission based on Berreman modes of Al_2_O_3_ and Si_3_N_4_. Additionally, the transparent materials ensure a high visible transmittance (∼84 %) of DRCG. We have developed a calculation model that accounts for the presence of neighboring objects around the wall-mounted cooler. Both the calculation and experimental results have confirmed that our DRCG significantly enhances cooling performance (>1.5 °C) compared with C-glass in hot urban environments characterized by neighboring object temperatures of 60 °C and a sky view factor of 0.25. In addition to its remarkable surface cooling capabilities, the enclosure cooling experiments have demonstrated the enhancement of space-cooling performance by ∼1.5 °C. With its outstanding capability to passively cool enclosures when used as a window, our DRCG offers a promising solution by effectively dissipating the trapped heat within urban areas to the external heat sink, thereby reducing energy consumption for cooling purposes. Furthermore, the high transmittance and directional thermal emission properties present opportunities for alternative applications, such as a display film that enhances thermal comfort for users positioned in front of the display by preventing heat transfer to the users. For broader applications of our DRCG, further research is necessary to improve the absolute emissivity at high incident angles.

## 4 Methods

### 4.1 Optical simulation for spectral results

To simulate the transmission, reflection, and emissivity spectra of the materials (*i.e.*, soda lime glass and ITO) and the DRCG, we employed commercial software known as DiffractMOD, developed by Rsoft Design Group, Synopsys, USA. This software utilizes rigorous coupled wave analysis to provide accurate spectral results. In our simulations, we considered the complex refractive index dispersions for various materials, including Si_3_N_4_, Al_2_O_3_, ITO, and soda lime glass, to ensure the precision of our spectral data.

### 4.2 Fabrication of DRCG

A clean commercial ITO-coated glass was prepared. A deposition of 210 nm of Si_3_N_4_ was conducted by the plasma-enhanced chemical vapor deposition method via PECVD (System 100, Oxford, USA). Subsequently, a deposition of 230 nm of Al_2_O_3_ was performed by the electron beam evaporation method via an e-beam evaporator (KVE-E2000, Korea Vacuum Tech Ltd, Korea). The deposition rate and pressure were approximately 1 Å s^−1^ and 10^−6^ Torr, respectively.

### 4.3 Structural and spectral analyses of the samples

The absorptivity spectra were characterized using an integrating sphere, encompassing a wavelength range from 280 to 2500 nm, and employing an ultraviolet–visible–near-infrared (UV–vis–NIR) spectrometer (Lambda 950, Perkin Elmer, Inc., USA). Additionally, the emissivity spectra for the MIR regions were analyzed using a Fourier-transform infrared spectrometer (VERTEX 70v, Bruker, USA), equipped with a variable angle reflection accessory. The emissivity spectra were determined based on the measured reflectance and transmittance spectra (*i.e.*, *E* = 100 % – *R* – *T*). To observe the cross-section of the fabricated sample, SEM was utilized, with a specific instrument being the S-4700 model from Hitachi Hi-Technologies, Japan.

### 4.4 Thermal equilibrium equation

The revised thermal equilibrium equation, as shown in Equation (3), is composed of the following six terms:

(4) $$\begin{array}{ll} P_{\text{rad}}\left(T_{\text{sample}}\right) & =\int_{0}^{\pi }\int_{0}^{\pi }\int_{0}^{\infty }I_{\text{BB}}\left(T_{\text{sample}},\lambda \right)\varepsilon^{\prime }\left(\lambda ,\theta \right)\\ & \;\times \cos\left(\theta \right)\sin\left(\theta \right)\text{d}\lambda d\theta d\phi , \end{array}$$

(5) $$P_{\text{Sun}}=A\left(\alpha \right)\cos\left(\alpha \right)\int_{0}^{\infty }I_{\text{AM}1.5\text{G}}\left(\lambda \right)\varepsilon \left(\lambda ,\alpha \right)\text{d}\lambda ,$$

(6) $$\begin{array}{ll} P_{\text{amb}} & =\int_{0}^{\pi }\int_{0}^{\theta_{1}}\int_{0}^{\infty }I_{\text{BB}}\left(T_{\text{amb}},\lambda \right)\varepsilon^{\prime }\left(\lambda ,\theta \right)\varepsilon_{\text{amb}}\left(\lambda ,\theta \right)\cos\left(\theta \right)\\ & \;\times \sin\left(\theta \right)\text{d}\lambda d\theta d\phi +R_{\text{ground}}\int_{0}^{\pi }\int_{\theta_{3}}^{\pi }\int_{0}^{\infty }I_{\text{BB}}\\ & \;\times \left(T_{\text{amb}},\lambda \right)\varepsilon^{\prime }\left(\lambda ,\theta \right)\varepsilon_{\text{amb}}\left(\lambda ,\theta \right)\times \cos\left(\theta \right)\sin\left(\theta \right)\text{d}\lambda d\theta d\phi , \end{array}$$

(7) $$\begin{array}{ll} P_{\text{nb}} & =\int_{0}^{\pi }\int_{\theta_{1}}^{\theta_{2}}\int_{0}^{\infty }I_{\text{BB}}\left(T_{\text{nb}},\lambda \right)\varepsilon^{\prime }\left(\lambda ,\theta \right)\varepsilon_{\text{nb}}\left(\lambda ,\theta \right)\\ & \;\times \cos\left(\theta \right)\sin\left(\theta \right)\text{d}\lambda d\theta d\phi +R_{\text{ground}}\int_{0}^{\pi }\int_{\theta_{2}}^{\theta_{3}}\int_{0}^{\infty }I_{\text{BB}}\\ & \;\times \left(T_{\text{nb}},\lambda \right)\varepsilon^{\prime }\left(\lambda ,\theta \right)\varepsilon_{\text{nb}}\left(\lambda ,\theta \right)\cos\left(\theta \right)\sin\left(\theta \right)\text{d}\lambda d\theta d\phi , \end{array}$$

(8) $$\begin{array}{ll} P_{\text{ground}} & =\int_{0}^{\pi }\int_{\theta_{2}}^{\pi }\int_{0}^{\infty }I_{\text{BB}}\left(T_{ground},\lambda \right)\varepsilon^{\prime }\left(\lambda ,\theta \right)\varepsilon_{\text{ground}}\left(\lambda ,\theta \right)\\ & \;\times \cos\left(\theta \right)\sin\left(\theta \right)\text{d}\lambda d\theta d\phi , \end{array}$$

(9) $$P_{\text{non }-\text{ rad}}=h_{c}\left(T_{\text{sample}}-T_{\text{ambient}}\right).$$

Here, *θ* and *ϕ* represent the zenith angle and the azimuth angle, respectively. The term $\varepsilon^{\prime }\left(\lambda ,\theta \right)$ is the averaged emitter’s emissivity over the azimuth angle in the zenith direction, corresponding to $\frac{1}{\pi }\int_{0}^{\pi }\varepsilon \left(\lambda ,\theta ,\phi \right)\text{d}\phi ,$ where *ɛ*(*λ*, *θ*, *ϕ*) is the spectral emissivity of the emitter with dependencies on zenith and azimuth angles (Figure S10). The azimuth angles are defined in the range of 0 to *π*, excluding angles toward the wall (*ϕ* = *π* to 2*π*) due to the absence of emissivity. $I_{\text{BB}}=\left(2h_{c}c^{2}/\lambda^{5}\right)/\left[e^{\frac{h_{c}}{\lambda k_{B}T}}-1\right]$ is the spectral radiance of a blackbody at temperature *T*, where *h, c, k*_*B*_ *,* *λ* *,* and *h*_*c*_ are Planck’s constant, the velocity of light, Boltzmann constant, wavelength, and the non-radiative heat exchange coefficient, respectively. *A*(*α*) is the constant that depends on the solar altitude angle *α*, as defined in Figure S18. The terms *ε*(*λ, α*) and cos(*α*) in Equation (5) represent the spectral emissivity of the emitter at incident angle *α*, and the orthogonal projection of the sunlight onto the vertical surface, respectively. We defined the solar altitude angle as 75°, typical summer season conditions in Gwangju, South Korea (35.166″N, 126.916″E) [52]. *R*_ground_ is the spectral reflectivity of the ground. The atmospheric emissivity is expressed as $\varepsilon_{\text{amb}}\left(\lambda ,\theta \right)=1-t{\left(\lambda \right)}^{1/\cos\left(\theta \right)}$ , where *t* is the sky transmission of mid-latitude atmospheric transmission in summer, calculated utilizing MODTRAN 6. *θ*_1_, *θ*_2_, and *θ*_3_ are the boundary angles of the four different situations for heat absorption to the glass (Figure S11). The emissivity of the neighboring objects and ground is expressed as $\varepsilon_{\text{nb}}\left(\lambda ,\theta \right)=\varepsilon_{\text{nb,0}}\cos\left(\frac{\pi }{2}-\theta \right)$ and $\varepsilon_{\text{ground}}\left(\lambda ,\theta \right)=\left(1-R_{\text{ground}}\right)\cos\left(\pi -\theta \right)$ , respectively, as considered Lambertian emission. *ɛ*_nb,0_ is assumed as 1 due to the high emissivity of the building exteriors [47]. The schematic for Lambertian emission of the neighboring objects and ground with angular property appears in Figure S19.

### 4.5 Measurements of thermal imaging and cooling performance

For thermal imaging purposes, the fabricated DRCG was positioned on a hot plate set to 60 °C. A thermal camera (E6, FLIR Systems, Inc., USA) was used to capture sample surface images at various incident angles. To facilitate real-time temperature measurements, temperature sensors (ST-50, RKC Instrument Inc., Japan) were employed. The tolerance of the sensors was measured within 0.1 °C (Figure S20). These sensors were affixed to the back side of the glass and the black material (*i.e.*, leather) inside the miniature houses to measure the surface and enclosure temperatures, respectively. Additionally, temperature sensors were inserted between the electrical heater (KHLVA-202/10-P, Omega Engineering, USA) and the black leather to monitor the heater temperature. All sensors were connected to a data logger (RDXL6SD, Omega Engineering, USA). Furthermore, an ambient air temperature sensor was used to measure the temperature of the naturally convective air.

## Supplementary Material

Supplementary Material Details
