# Supplementary material for: Transparent energy-saving windows based on broadband directional thermal emission
Source: Nanophotonics. 2024 Jan 9;13(5):749–61. doi: 10.1515/nanoph-2023-0580 (PMC11501872; doi:10.1515/nanoph-2023-0580)
Supplement: Supplementary file 1 — Supplementary Material Details [file j_nanoph-2023-0580_suppl_001.docx]

Supplementary Material

Transparent energy-saving windows based on broadband directional thermal emission

Minyeol Bae^1^, Do Hyeon Kim^1^, Sun-Kyung Kim^2,*^, and Young Min Song^1, 3*^

^1^ School of Electrical Engineering and Computer Science, Gwangju Institute of Science and Technology, Cheomdangwagi-ro 123, Buk-gu, Gwangju 61005, Republic of Korea

^2^ Department of Applied Physics, Kyung Hee University, Gyeonggi-do 17104, Yongin, Republic of Korea

^3^ Artificial Intelligence (AI) Graduate School, Gwangju Institute of Science and Technology, Cheomdangwagi-ro 123, Buk-gu, Gwangju 61005, Republic of Korea

*Corresponding author email: [sunkim@khu.ac.kr](mailto:sunkim@khu.ac.kr), [ymsong@gist.ac.kr](mailto:ymsong@gist.ac.kr)

**Fig. S1.** Calculation results of cooling temperature of ideal directional emitters as a function of *h_c_* values. We designed ideal directional emitters that radiate their heat only in the LWIR region and angle range of *θ*. As a result, only the ideal emitters with emission angles higher than 60˚ demonstrate the temperature reduction properties.

**Fig. S2.** Measured and simulated transmittance, reflectance over the vis-NIR region of soda lime glass.

**Fig. S3.** Optical measurements of the ITO. (A) Measured and simulated transmittance, reflectance over the vis–NIR region of the ITO. (B) Measured and simulated reflectance over the MIR region of the ITO.

**Fig. S4.** LWIR properties of materials. (A, B) Refractive indices over LWIR region of (A) soda lime glass and (B) ideal broadband ENZ.

**Fig. S5.** Optical constants of the materials. (A-C) Optical constants in solar spectrum (left) and thermal range (right) of (A) Al_2_O_3_ [1] (B) Si_3_N_4_ [2], and (C) ITO [3].

**Fig. S6.** Calculated emissivity spectrum varying with the incident angle and wavelength of the three structures with different thicknesses in p-polarization. The dashed lines indicate 53˚ (left), 62˚ (middle), and 75˚ (right), the peak angle of each structure.

**Fig. S7.** DRCG with insulator-metal-insulator (IMI) structure to enhance NIR reflectance. (A) The schematic of the DRCG incorporating IMI structure. The IMI structure consists of TiO_2_ (30 nm)/Ag (15 nm)/TiO_2_ (30 nm). (B) Transmittance and Reflectance over visible and Vis-NIR spectrum of the DRCG with IMI structure. (C) LWIR emissivity spectra of the DRCG with IMI structure.

**Fig. S8.** Calculated results of cooling temperature of directional emitters as a function of non-radiative heat exchange coefficient (*h_c_*) values. We designed the directional emitters for LWIR emission within the angle range of 60˚ to 80˚. As a result, emitters with emissivity higher than 20% enable lowering the temperature than ambient temperature.


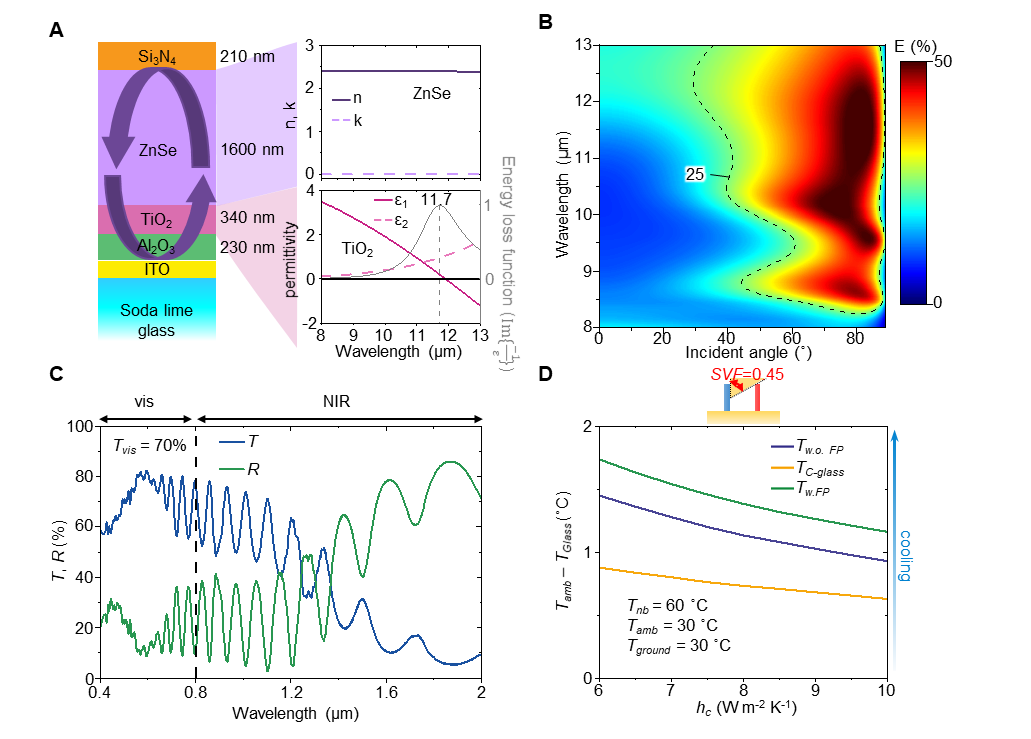


**Fig. S9.** DRCG with ZnSe gap. (A) Structure of DRCG with Fabry-Pérot (FP) resonance (left) and optical data of the materials (right). The optical constants are obtained from previously published research [4, 5]. (B) Calculated emissivity spectrum of the DRCG with FP resonance varying with the incident angle and wavelength in p-polarization. (C) Calculated vis-NIR transmittance and reflectance of the DRCG with FP resonance. (D) Calculated cooling performance of the DRCG with FP resonance compared to the original DRCG (without FP resonance) and the C-glass as a function of the non-radiative heat exchange coefficient (*h_c_*).

**
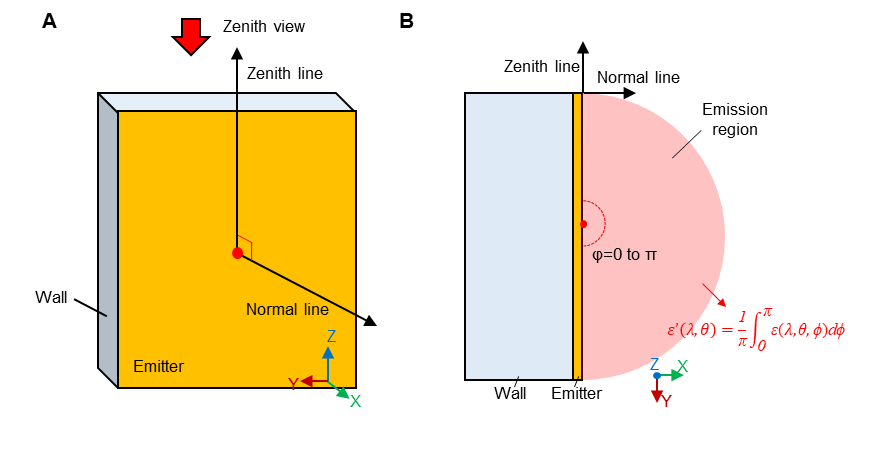
**

**Fig. S10.** The averaged emissivity over the azimuth angle. (A) Schematic of the zenith line and the emitter’s normal line. (B) Illustration of the emission region (0 to π) at the zenith view.


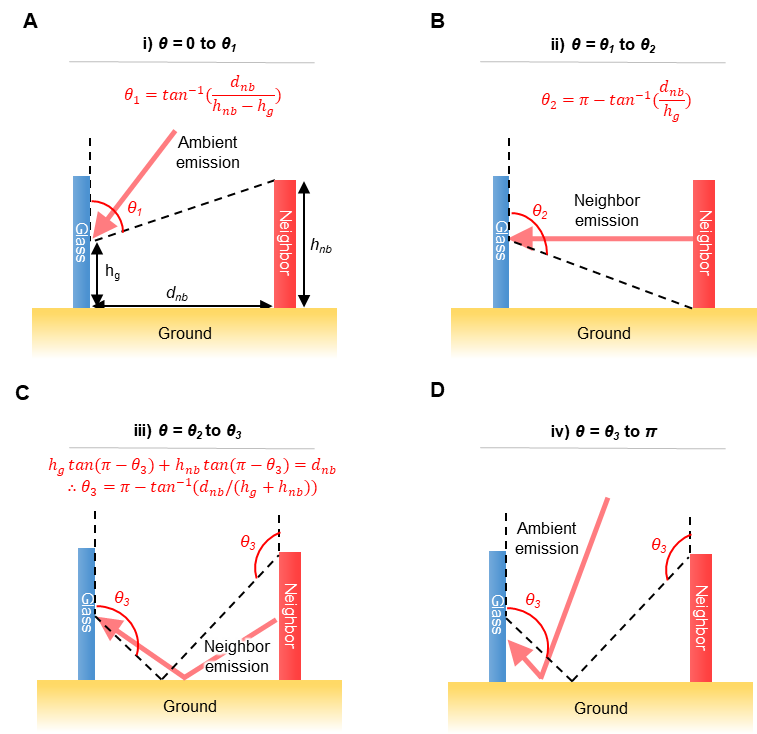


**Fig. S11.** Four different situations for heat absorption to the glass. (A) Ambient emission within the theta from 0 to *θ_1._* (B) Neighbor emission within the theta from *θ_1_* to *θ_2._* (C) Neighbor emission within the theta from *θ_2_* to *θ_3._* (D) Ambient emission within the theta from *θ_3_* to π.

**Fig. S12.** Calculated SVF (A) of the window and (B) the ground [6].

**Fig. S13.** Comparison of the low-emissivity (Low-E) glass and the DRCG. (A) Transmittance and reflectance over the visible and NIR spectrum of Low-E glass. The Low-E glass is composed of ZnS (31 nm)/Ag (20 nm)/ZnS (34 nm) on a glass substrate [7]. Due to the thin silver layer, the Low-E glass exhibits a lower visible transmittance (76%) than our DRCG (84%). (B) Simulated temperature difference between the DRCG and C-glass with different ground temperatures (*T_ground_*) and thermal ground albedo (*R_ground_*).


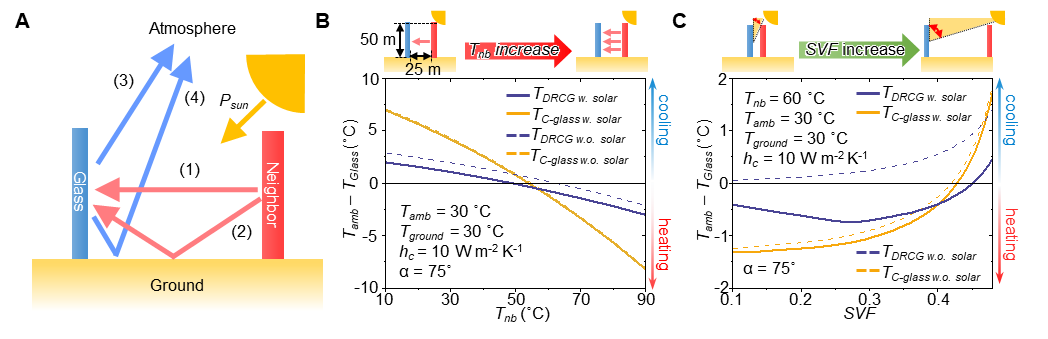


**Fig. S14.** Theoretical surface cooling performance of the DRCG and C-glass under the sunlight. (A) Schematic of the heat exchanging process among the neighboring objects, glass, and Sun. (B, C) Calculated cooling temperature of the DRCG and the C-glass as a function of the (B) neighboring object temperature and (C) SVF.


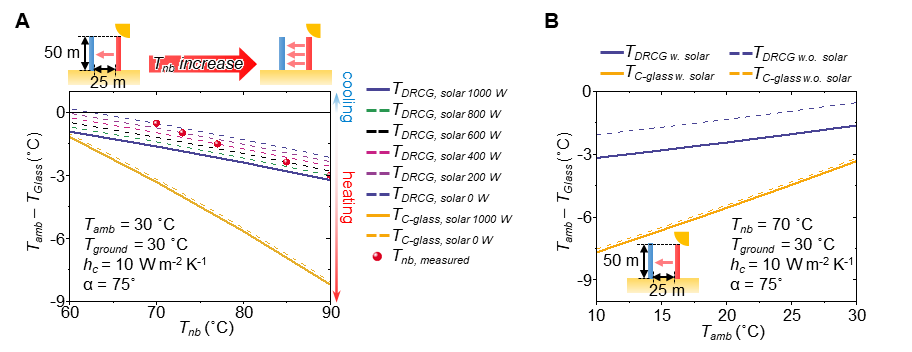


**Fig. S15.** Theoretical surface cooling performance of the DRCG and C-glass with different solar irradiance and the ambient temperature. (A) Simulated cooling temperature of the DRCG and the C-glass as a function of the neighboring object temperature (*T_nb_*) with different solar intensity. The red points are the measured neighboring object temperatures at different solar intensity from Fig. S16. (B) Calculated cooling temperature of the DRCG and the C-glass as a function of the ambient temperature (*T_amb_*).

**Fig. S16.** Surface cooling performance of the DRCG and the C-glass with different heater temperatures under sunlight. (A) Schematic and photograph of the surface cooling measurement setup. (B) Experimental result of surface temperatures of the DRCG and the C-glass with different heater temperatures.

**Fig. S17.** Surface and enclosure cooling performance of the DRCG and C-glass under sunlight. (A) Schematic and photograph of the surface cooling measurement setup. (B) Experimental result of surface temperatures of the DRCG and the C-glass. (C) Illustration and optical image of the enclosure cooling measurement setup. (D) Experimental result of the enclosure temperature of the DRCG and C-glass.
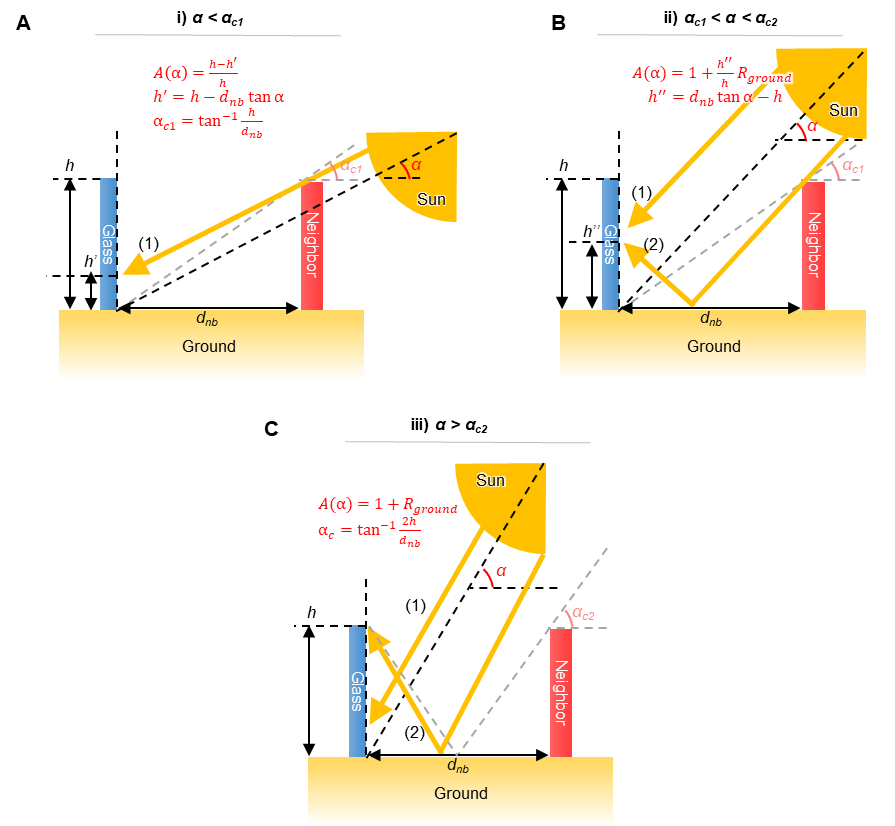


**Fig. S18.** Solar radiation to the glass in different situations. The solar altitude angle (A) lower than *α_c1_* (*i.e.*, *α* < *α_c1_*), (B) between *α_c1_* and *α_c2_* (*i.e.*, *α_c1_* < *α* < *α_c2_*), and (C) higher than *α_c_* (*i.e.*, *α* > *α_c2_*).


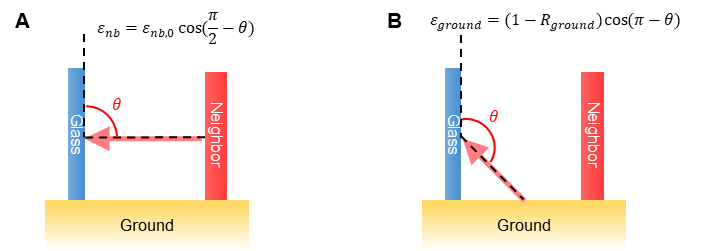


**Fig. S19.** Lambertian emission of the surrounding objects (*i.e.*, ground and neighboring object). (A) Lambertian emission of the neighboring objects at 0 < *θ < π,* can be expressed as $\varepsilon_{nb,0}\cos(\frac{\pi}{2}-\theta)$*.* (B) Lambertian emission of the ground at *π/2* < *θ < π,* can be expressed as $(1-R_{ground})cos (\pi-\theta)$.

**Fig. S20.** Accuracy of thermocouple sensors. (A) Photograph of the experimental setup. (B) Experimental results of the temperature difference between two thermocouples. The measured tolerance was within 0.1 ˚C.

**References**

[1] M. R. Querry, “Optical constants,” https://apps.dtic.mil/sti/citations/ADA158623 (retrieved Oct. 17, 2023)

[2] J. Kischkat, S. Peters, B. Gruska, et al., "Mid-infrared optical properties of thin films of aluminum oxide, titanium dioxide, silicon dioxide, aluminum nitride, and silicon nitride," *Appl. Opt.*, vol. 51, no. 28, pp. 6789-6798, 2012.

[3] R. Kamakura, T. Takeishi, S. Murai, K. Fujita, and K. Tanaka, "Surface-enhanced infrared absorption for the periodic array of indium tin oxide and gold microdiscs: Effect of in-plane light diffraction," *ACS Photonics*, vol. 5, no. 7, pp. 2602-2608, 2018.

[4] D. T. F. Marple, "Refractive index of ZnSe, ZnTe, and CdTe," *J. Appl. Phys.*, vol. 35, no. 3, pp. 539-542, 2004.

[5] D. Franta, D. Nečas, and I. Ohlídal, "Universal dispersion model for characterization of optical thin films over a wide spectral range: application to hafnia," *Appl. Opt.*, vol. 54, no. 31, pp. 9108-9119, 2015.

[6] N. E. Theeuwes, G.-J. Steeneveld, R. J. Ronda, and A. A. M. Holtslag, "A diagnostic equation for the daily maximum urban heat island effect for cities in northwestern Europe," *Int. J. Climatol.,* vol. 37, no. 1, pp. 443-454, 2017.

[7] A. M. Al-Shukri, "Thin film coated energy-efficient glass windows for warm climates," *Desalination,* vol. 209, no. 1, pp. 290-297, 2007.
